# Biocompatibility of a New Antibacterial Compound and its Effect on the Mechanical Properties of Flowable Dental Composites (Animal Study)

**DOI:** 10.30476/dentjods.2019.77836.0

**Published:** 2020-03

**Authors:** Mehdi Abaszadeh, Meisam Mohammadi, Iman Mohammadzadeh

**Affiliations:** 1 Pharmaceutics Research Center, Institute of Neuropharmacology, Kerman University of Medical Sciences, Kerman, Iran; 2 Dept. of Mechanical Engineering, Vali-e-Asr University of Rafsanjan, Rafsanjan, Iran; 3 Oral and Dental Disease Research Center, Kerman University of Medical Sciences, Kerman, Iran

**Keywords:** Pyrazole, Antibacterials agents, Streptococcus mutans, Dental Materials, Dental Caries, Biocompatibility

## Abstract

**Statement of the Problem:**

Recently, new compound of 3, 5-dimethyl-1-thiocarboxamide pyrazole has been composed with excellent antibacterial property. Biocompatibility and its effects on mechanical properties of dental composites should be considered before clinical use.

**Purpose:**

The purpose of this study was to evaluate the biocompatibility of 3, 5-dimethyl-1-thiocarboxamide pyrazole as a new antibacterial compound and its effect on the mechanical properties of dental composites.

**Materials and Method:**

In this experimental study, a new antibacterial compound was synthesis by reaction between Thiosemicarbazide and 2, 4-Pentandione and tested on thirty male albino Wistar rats weighting 200-250gr. Rats were randomly divided into 3 groups of 10, each rat received 3 implants of 3,5-dimethyl-1-thiocarboxamide pyrazole, penicillin v and empty polyethylene tube. A pathologist, who was unaware of types of tested materials and timing, performed the examination of specimens. The depth of cure and flexural strength of resin composite was measured using Iso4049 standard technique. Compressive strength was determined according to Iso9917 standard.

**Results:**

This compound was biocompatible and there was no significant difference in flexural strength and compressive strength of the composites containing 1% of this compound with the control group (*p*> 0.05).

**Conclusion:**

The 3, 5-dimethyl-1-thiocarboxamide pyrazole with a concentration of 1% in flowable composites can be very effective in preventing secondary caries.

## Introduction

Todays, dental composites have attracted many patients and dentists regarding their beauty and the convenience of their application [ 1 ]. Despite of the great progresses achived in these dental materials, one of the biggest problems that attracted the attention of many researchers is the weakness of these compounds against bacteria. This problem reduces their useful life and ultimately leads to substitution. Bacteria are a complex poly-microbial population that tends toward composites’ surface, as the bacteria's tendency for these compounds is more than tooth enamel [ 1 - 6 ]. The accumulation of these bacteria on the composite surface leads to secondary caries. In recent years, many studies have focused on creating antibacterial properties in composites [ 1 - 2 ]. One way to achieve this goal was to use the combination of 3,5-dimethyl-1-thiocarboxamide pyrazole [ 7 ].

Using this compound, a very good antibacterial property was obtained in flowable composites [ 7 ]. It should be noted that considering only antibacterial properties in composites are not efficient in dental restoration and should be biocompatible with suitable mechanical properties. In this study, tissue compatibility of 3, 5-dimethyl-1-thiocarboxamide pyrazole was studied. In order to evaluate the biological response due to the new dental material, in vivo methods such as implantation of these substances in subcutaneous tissue of laboratory animals were studied first [ 8 - 9 ].

Therefore, in this study, the tissue biocompatibility assessment of the composition of 3,5-dimethyl-1-thiocarboxamide pyrazole was investigated in rat subcutaneous tissue. Any restorative material should have good mechanical properties of tooth as well as good biocompatibility in order to be able to use it in tooth restoration. The mechanical properties of composites containing the aforementioned compound were also investigated.

## Materials and Method

### Synthesis of 3, 5-dimethyl-1-thiocarboxamide pyrazole

**Figure1:**
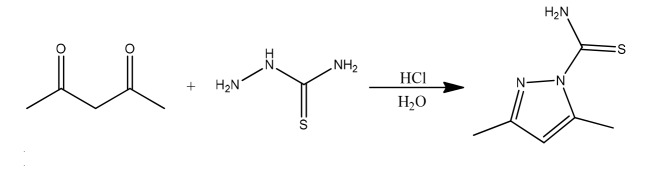
Synthesis of 3, 5-dimethyl-1-thiocarboxamide pyrazole

3, 5-dimethyl-1-thiocarboxamide pyrazole was synthesized by reaction between thiosemicarbazide and 2, 4-Pentandione with a molar ratio of one to one. 5 gr of thiosemicarbazide was dissolved in 250 ml cold water and 1ml of concentrated hydrochloric acid. Prepared liquid was filtered using filter paper and 5ml of 2, 4-Pentandione was added and stirred for 30 minutes. Passing one hour of stirring, a white precipitate, 3, 5-dimethyl-1-thiocarboxamide pyrazole was formed (See Figure 1) and purified using ethanol [ 10 ].

### Biocompatibility 

In the present animal study, thirty male albino Wistar rats weighting 200-250 gr were used. The experiment was performed in accordance to “Principles of laboratory animal care” formulated by National Society for Medical Research and “Guide for the care and use of laboratory animals” prepared by Institute of Laboratory Animal Resources, National Research Council and published by the National Academy Press, revised 1996. Veterinarian performed rats’ anesthesia via the peritoneal method using 47.5 mg kg-1 of Ketamine hydrochloride and 0.01 mg kg-1 of Rompun 2%. For each animal, the hair was cut from the back of the animal to the right and left (two anterior regions and one posterior region) with 10% scarlet brown iodine. All regions were disinfected using green iodine and in each region a small incision with a length of approximately 12 mm was made by a razor scalpel number 20. Subsequently, using a blunt dissecting backside, a 20-mm bundle of subcutaneous tissue was cut for implanting tubes.

Rats were randomly divided into 3 groups of 10, each rat received 3 implants of 3,5-dimethyl-1-thiocarboxamide pyrazole, penicillin v, and empty polyethylene tube. Then, it was sutured plainly and individually with nylon yarn. The application of penicillin-v was because of its current use as a routine antibiotic. One week, one month and two months after the tubes were implanted [ 11 ]; the rats were sacrificed by injecting too much ketamine HCl. The hair extensions of the tubes were re-shaved, the skin and connective tissue surrounding the implant were brought out the block section and kept in the formalin 10% for at least 48 hours. After fixing, parallel sections with longitudinal axis and tissue were prepared for staining with Hematoxylin Eosin. A pathologist, without knowing the type of tested material and timing, performed the analysis of samples. To evaluate the histological response, studies of Stanford [ 11 ] and Yaltirik *et al*. [ 12 ], Ozbas *et al*. [ 13 ] and Derakhshan *et al*. [ 14 ] were used. 

### Histological criteria

The tissue reaction at both ends of the tubes was studied according to the following histological criteria: The size of the subcutaneous tissue around the tube was measured at 100 magnifications and in micrometers. The score were defined as (0) for no capsules, (1) when the thickness of the capsule was less than 150 μm, (2) when the thickness of the capsule was greater than 150 μm. The severity of inflammation was defined by observing the number of inflammatory cells in or around the subcutaneous tissue capsule with a magnification of 40 fold and scored as (0) for no inflammation, (1) for less than 25 cells, (2) between 25 and 50 cells, (3) between 50 and 75 cells, (4) for more than 75 cells.

For the extent of inflammation , the amount of expansion of inflammatory cells at both ends of the tube was observed at 40x magnification and scored as (1) when Inflammatory cells only appeared in the surface layer of the capsule,(2) when inflammatory cells were limited to fibrous capsules, (3) when inflammatory cells were seen around the capsule. For detecting necrosis, the disappearance of cells or the observation of the ghost of cells without a nucleus was observed with 40x magnification and scored as (0) for absence of necrosis, and (1) for presence of necrosis.

For assessing the inflammation type, different types of inflammation were defined according to the related inflammatory cells as acute inflammation (p (polymorphonuclear cells) and chronic inflammation (macrophages, plasma cells and lymphocytes) with the score of (0) for no inflammation, (1) for chronic inflammation, and (2) for acute inflammation. According to the available data presented in the literatures, 10 samples were utilized for each period [ 13 - 16 ].

### Data analysis

By describing the findings, non-parametric methods such as Kruskal-Wallis were used based on the rank of the variables. Mann-whitney U test was applied for comparing the two methods and the significant correlation was modified with Bonferroni method.

### Preparation of composites containing 3, 5-dimethyl-1- thiocarboxamide pyrazole

Six groups of the specimen were prepared by mixing of 3, 5-dimethyl-1-thiocarboxamide pyrazole with resin composite (Tetric flow, Ivoclar Vivadent, USA) in 1, 2, 3, 4 and 5 wt% and 0 wt% as the control group. Mixing was performed in a dark room for 15 min by spatula [ 17 ].

### Measurement of the depth of cure

The depth of cure of composite’s resin was measured using Iso4049 standard technique. The dental composite resin was placed inside a cylindrical mold (with 10 mm depth and 4 mm diameter) and cured by using a light cure instrument (LED, DEML, SDS Kerr, USA, with an intensity of circa 800 mW cm-2) from above for 40 seconds. Upon completion of the exposure, the cured composite was removed from the mold and uncured material was separated from the end of composite with the spatula, and then by using the digital caliper, the height of the cured resin was measured and finally, this value was divided into two and was reported as the depth of the cure [ 17 ]. The test was repeated three times and the mean of the obtained values was reported.

### Flexural strength measurement

Flexural strength is one of the most important mechanical tests to determine the efficiency of dental resins. In accordance with Iso4049 standard, the resin was poured in a rectangular cube mold with a dimension of 25x2x25mm. The filled mold was closed by glass slides at the ends and was cured using a light cure instrument for 40 seconds through the both ends. The cured specimens were removed from the mold and put in deionized water at 37°C for 1 day. Later, both surfaces of the samples are polished in a wet environment. For each formulation, 10 samples were tested. The three-point bending test was performed using M350-10CT Testometric device at the rate of 0.5 mm / min. Finally, bending strength (Fs) was calculated using the following relation.

$\mathrm{Fs}=\frac{\mathrm{3pl}}{{\mathrm{2bd}}^{2}}$

In the above relation, p, l, b and d present resistance at break point, distance between the two locations of the sample on the device (20 mm), sample width and sample thickness, respectively.

### Compressive strength measurement

According to Iso9917 standard for compressive strength tests, the sample was located in a cylindrical metal frame with a diameter of 4 mm and a height of 6 mm. Prepared sample was cured for 40 seconds through both ends and put in 37°C water for 24 hours. The finalized samples were subjected to pressure using M350-10CT Testometric device at the rate of 1mm/min. Ten samples of each formulation were tested [ 15 ].

### Data analysis

The resulted mechanical test data were analyzed by one-way ANOVA, and the Tukey post hoc HSD multiple comparison test. The level of significance was considered as *p*= 0.05.

## Results

### Evaluation of histopathologic observations in considered groups

#### Case 1: Empty polyethylene tube group

The capsule thickness was increased during the time, so that during 7 days, growth in thickness of the capsule was less than 30 days. Moreover, it was observed that the thickness growth of capsule was more significant after 60 days in comparison with 7days (*p*< 0.05).

Severity of inflammation, extent of inflammation, and presence of chronic inflammation were significantly higher in the 7-day interval than 30-day and 60-day periods (*p*< 0.05). However, no significant difference was observed between 30 and 60 days (*p*> 0.05).

Acute necrosis and inflammation were not observed in any of the periods.

#### Case 2: 3, 5-dimethyl-1-thiocarboxamide pyrazole group

In this case, thickness of the capsule increased over the time, although this difference was not significant (*p*> 0.05).The extent of inflammation decreased by 7, 30 and 60 days, but there was no apparent difference between the groups. The severity of inflammation decreased by 7, 30, and 60 days, and a significant decrease was seen for the time duration of 60 days in comparison with the 7 days (*p*< 0.05).Acute necrosis and inflammation were not observed in any group.

#### Case 3: Penicillin V group

The thickness of the capsule in the 30 and 60 days’ periods was severely higher than the 7-days period. However, no clear difference was seen (*p*> 0.05). The severity of inflammation and the extent of inflammation after 60 days was less than 30 days and 7 days, but this difference was not statistically significant (*p*> 0.05). The same as other groups, acute necrosis, and inflammation were not recognized in any of the samples.

### Histological evaluation and comparison between the groups studied at different time periods

####  After seven days

There was no apparent difference in the amount of capsule thickness, intensity of inflammation and extent of inflammation between different groups during this time period (*p*> 0.05).

#### After thirty days

During this period, there was no significant variation in the thickness of the capsule, severity of inflammation, extent of inflammation and type of inflammation between the three different groups (*p*> 0.05).

#### After sixty days

**Figure2:**
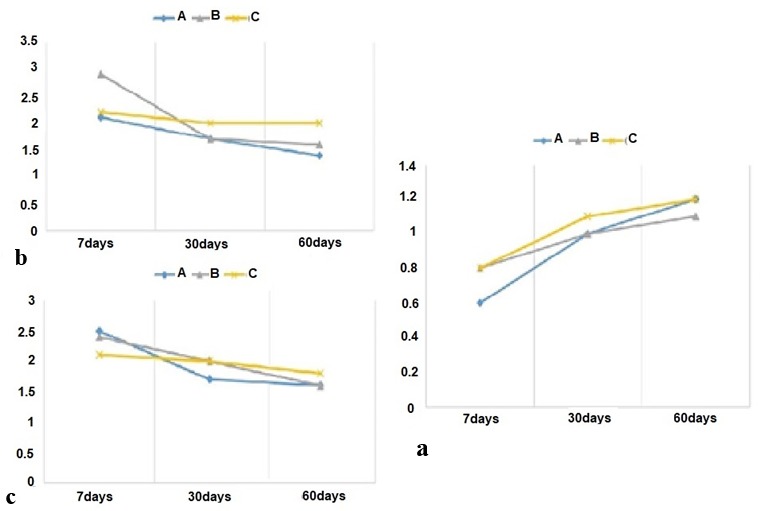
**a:** Thickness of subcutaneous tissue capsule in various time intervals, **b:** Severity of inflammation in different time intervals, **c:** Variation of extent of inflammation versus time. (A: Empty polyethylene tube, B: 3, 5-dimethyl-1-thiocarboxamide pyrazole, C: Penicillin V)

In this period, there was no clear difference in any cases of capsule thickness, intensity of inflammation, extent of inflammation and type of inflammation between 3 different groups (*p*> 0.05). Results are shown in Figure 2.

#### Depth of cure

**Figure3:**
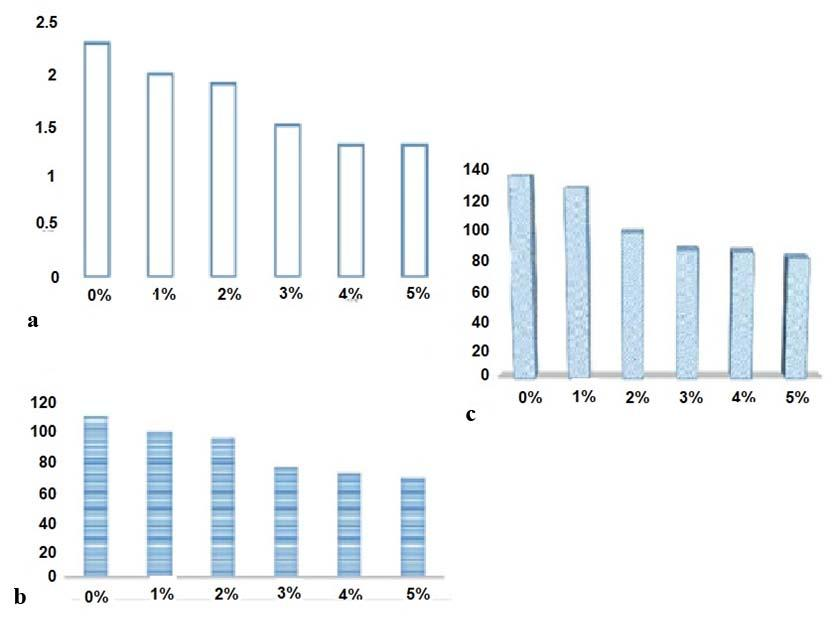
**a:** Depth of cure of flowable composites containing 3, 5-dimethyl-1-thiocarboxamide pyrazole, **b:** Flexural strength of flowable composites containing 3, 5-dimethyl-1-thiocarboxamide pyrazole, **c:** Compressive strength of flowable composites containing 3, 5-dimethyl-1-thiocarboxamide pyrazole

It was seen that by increasing the percentage of composition of 3,5-dimethyl-1-thiocarboxamide pyrazole, the depth of cure decreases, so that there was no certain change between control group and 1% and 2% groups (*p*> 0.05). However, there was significant difference between control group and 3%, 4% and 5% groups, (*p*< 0.05). Results are depicted in Figure 3.

#### Flexural strength 

By increasing the percentage of 3,5-dimethyl-1-thiocarboxamide pyrazole, the flexural strength increased so that there was no visible change between the control group and the 1% and 2% groups (*p*> 0.05). However, a significant difference with the rest of the groups was present (*p*< 0.05) (Figure 3).

#### Compressive strength

Increasing the composition of 3, 5-dimethyl-1-thiocarboxamide pyrazole, decreased the compressive strength, where no statistically significant difference between the control group and the 1% group was seen, *p*> 0.05, while the other groups had a significant variation in comparison with the control group (*p*< 0.05) (Figure 3).

## Discussion

The success of antibacterial tests per se does not indicate the usefulness of these substances for clinical use. In addition to antibacterial characteristic, tissue biocompatibility and suitable mechanical properties are also important factors.

To use any substance on humans, tissue biocompatible tests should be done. There are currently four methods for measuring the biocompatibility of materials. These methods include cell evaluation in the culture medium, subcutaneous implantation, intrabony implantation, and *ex vivo* evaluation of periradicular tissue in human specimens [ 16 ].

Subcutaneous implantation method was utilized in the present study where the substance was placed in polyethylene and was implanted in subcutaneous connective tissue. This method is one of the most common methods for assessing the relative adaptability of dental materials, introduced by Torneck [ 18 ]. The effectiveness of this method was re-evaluated and confirmed by Olson *et al*. [ 19 ]. They showed that insertion of the investigated material in the final parts of the polyethylene tubes prevents the release of material into the connective tissue, so this method has more advantages than the direct method of injecting into the connective tissue. Makkes *et al*. [ 20 ], Safavi *et al*. [ 21 ], Kim *et al*. [ 22 ] and Bachmann *et al*. [ 23 ] used polyethylene tubes for implanting. These tubes form very little stimulation in the surrounding tissue, or do not produce any reaction in the surrounding tissue. These are easy to use and to implant, and it seems to have a stable chemical structure not affected by the substances or agents inside the tube [ 20 ]. Tubes should be selected with a small diameter to minimize the possibility of displacement and excessive movement of the material. A length of 7-5 mm of tube is good enough to compare the surface of the materials tested at the ends of the tubes, which are in direct contact with tissue [ 22 ]. In the present study, tubes with 5 mm length and 1.7 mm diameter were applied with two open ends.

Stanford [ 11 ] considered two periods of time to investigate the adaptive response but Olsson *et al*. [ 19 ] suggested three periods of time. Olsson’s study was designed in 3 periods of 7, 30 and 60 days.

There are several methods to compare the severity of the inflammatory response around the tubes containing the observing substance. According to Stanford method [ 11 ], the number of inflammatory cells is counted in different regions of the microscopic section [ 20 ]. Since the inflammatory response of the connective tissue has different aspects, focusing solely on the number of inflammatory cells does not reflect all aspects of the connective tissue response and does not appear to be comprehensive [ 24 ].

Therefore, method proposed by Yaltirik *et al*. [ 12 ], was used so that in addition to the number and extent of inflammatory cells, other aspects of inflammation, such as the formation of fibrous capsules and the presence of necrosis were included. Since in similar studies, the type of inflammatory cells was studied [ 13 - 14 , 24 - 25 ], this variable was also considered in the present study.

According to the results of this study, in the 30-day and 60-day periods, in most samples of each of the three tested materials, a fibrous capsule was formed around the tube, which suggested good tissue tolerance of these materials. Formation of fibrous capsule around the material can keep it from the surrounding tissue and prevents damage of the material in the tissue [ 11 , 26 - 27 ]. Formation of a fibrous capsule was reported in many similar studies [ 11 - 12 , 27 - 29 ].

 According to the above studies, it was specified that the presence of 3, 5-dimethyl-1-thiocarboxamide pyrazole in flowable dental composites resulted in antimicrobial properties against Streptococcus mutans. It also had good biocompatibility, but the presence of this compound in flowable dental composites reduced mechanical properties of composites. This effect was not significant in composites containing 1% of the 3, 5-dimethyl-1-thiocarboxamide pyrazole. 

## Conclusion

According to the investigations, it was concluded that the compound 3, 5-dimethyl-1-thiocarboxamide pyrazole with concentration of 1% in flowable composites is very effective in preventing secondary caries without reducing the mechanical strength.
